# Understanding Original Antigenic Sin in Influenza with a Dynamical System

**DOI:** 10.1371/journal.pone.0023910

**Published:** 2011-08-29

**Authors:** Keyao Pan

**Affiliations:** Department of Bioengineering, Rice University, Houston, Texas, United States of America; University of Hong Kong, Hong Kong

## Abstract

Original antigenic sin is the phenomenon in which prior exposure to an antigen leads to a subsequent suboptimal immune response to a related antigen. Immune memory normally allows for an improved and rapid response to antigens previously seen and is the mechanism by which vaccination works. I here develop a dynamical system model of the mechanism of original antigenic sin in influenza, clarifying and explaining the detailed spin-glass treatment of original antigenic sin. The dynamical system describes the viral load, the quantities of healthy and infected epithelial cells, the concentrations of naïve and memory antibodies, and the affinities of naïve and memory antibodies. I give explicit correspondences between the microscopic variables of the spin-glass model and those of the present dynamical system model. The dynamical system model reproduces the phenomenon of original antigenic sin and describes how a competition between different types of B cells compromises the overall effect of immune response. I illustrate the competition between the naïve and the memory antibodies as a function of the antigenic distance between the initial and subsequent antigens. The suboptimal immune response caused by original antigenic sin is observed when the host is exposed to an antigen which has intermediate antigenic distance to a second antigen previously recognized by the host's immune system.

## Introduction

An immune memory comes from a previous infection or vaccination, stores the information for antigen recognition, and is activated in a future infection by a related pathogen. Long-term immune memory has been observed in various pathogens including smallpox [1], malaria [2], hepatitis B [3], dengue [4], and influenza A [5]. By recognizing and rapidly eliminating the reinfecting pathogen, this long-lasting effect can permanently or temporarily prevent the reinfection of the host by some pathogens [6]. In some cases, this long-lasting effect can also reduce the severity, duration, or risk of the infection and symptoms [7]. Smallpox virus, also called variola virus, only propagates in humans and has a relatively low mutation rate [8]. In contrast, influenza A virus propagates in humans, pigs, and aquatic birds, with a higher mutation rate that is approximately 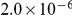/nucleotide/infectious cycle [9], or 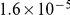/amino acid/day. Calculation of the binding free energy between human antibodies and circulating influenza A strains shows that the virus mutates away from the genotypes that code for hemagglutinin proteins well recognized by the human immune system [10]. Thus for influenza A, there is usually a significant antigenic distance between the circulating strain in a given year and the immune memory from previous years.

Original antigenic sin is the phenomenon in which prior exposure to an antigen leads to a subsequent suboptimal immune response to a related antigen [11]–[13]. In some years when the antigenic distances between vaccine and circulating virus strains fell into a certain range, the effect of original antigenic sin decreased the effectiveness of influenza vaccines. Historical data of influenza vaccines indicate that vaccine effectiveness does not monotonically decrease with the antigenic distance between the vaccine strains and the circulating strains, but rather has a minimum at an intermediate antigenic distance [14], [15]. Interestingly, since the vaccine effectiveness at this intermediate antigenic distance between the vaccine and circulating strains is lower than the effectiveness at a larger antigenic distance in unvaccinated people, original antigenic sin could make vaccinated people more susceptible to the virus than those who are unvaccinated.

The mechanism of original antigenic sin was previously studied using stochastic models at the cellular level [16], [17]. These previous studies developed stochastic models with thousands to millions of B cells [16], [17]. The stochastic models introduce various antigens to a repertoire of B cells. The B cells with higher affinity to an antigen have larger probability to be selected during the B cell maturation process. Earlier works discussing the mechanism of original antigenic sin at the cellular level include [17], which attributed original antigenic sin to the localization of the B cells in the secondary immune response around the B cells in the primary immune response in the amino acid sequence space. The affinity between an antibody and an antigen is given by the generalized 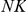 model (GNK model) of the three-dimensional protein structures [18]. The GNK model was derived from the 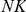 model which was originally introduced to model rugged fitness landscapes [19], [20] and evolutionary processes [21]–[23]. In the GNK model, the amino acid sequences of a group of influenza A specific antibodies are allowed to mutate freely and independently in the affinity landscape to maximize their affinities to the virus. B cells that produce antibodies with the highest affinities replicate into the next generation. The mutation of the virus is modeled by changing the fitness landscape. The antibody affinities at the end of the simulation correlate well with the vaccine effectiveness data observed in history [14].

The present study aims to use a set of ordinary differential equations (ODEs) to describe the interaction among the B cells, the virus particles, and the epithelial cells. This deterministic model can reduce the memory and CPU requirements, compared to the stochastic models [16], [17]. In this study, I developed a set of ODEs as the mean-field approximation of the stochastic models that store the information of each B cell. The ODEs give a deterministic explanation of original antigenic sin. This explanation agrees with both the observed data [14] and a previous explanation given by the GNK model [17]. Various ODE models have been established to describe and simulate the process of influenza A infection and the resulting immune response [24]–[29]. The basic elements of these ODE models were described in [30]. Automata have also been used to model the time-dependent spatial distribution of the tissue cells and the virus [31], [32].

In this study, I build a deterministic ODE-based model compatible with the previous GNK model [17] to reproduce the observed phenomenon of original antigenic sin. The main purpose of this paper is to address the following two questions on the modeling of original antigenic sin. First, can a deterministic dynamical model with a small number of state variables simulate the phenomenon of original antigenic sin, which was simulated by stochastic models of a large repertoire of B cells [16], [17]? Second, what is the mechanism of original antigenic sin revealed by the deterministic dynamical model? I address the first question by building a deterministic ODE model, which reproduces the phenomenon of original antigenic sin observed in experiment [11]–[13]. See the subsections Model Development and Description, Time Courses of Infection and Recovery, and A General Picture of Original Antigenic Sin in the section Results. I address the second question by analyzing the non-monotonicity of the overall effect of an immune response. See the subsection Mechanism of Original Antigenic Sin in the section Results. The values of the parameters mainly come from previous studies that simulated influenza infection and immune response and obtained plausible results. However, the limitations of the available experimental data do not allow one to develop an accurate model purely based upon experimental data [33]. Experimental data available for model development usually have limited quality or quantity, causing unavoidable overparameterization of the models. In the present dynamical model, two parameters 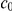 and 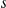 need to be estimated prior to the simulation. I give the estimation of parameters 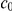 and 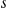 in the section Materials and Methods and perform the sensitivity analysis of both of them in the section Results. The terms in the ODEs have clear physical meanings, so my model explicitly illustrates the details of the influenza A infection and the immune response. A comparison between this deterministic dynamical model and previous stochastic models is presented in the section Discussion. A brief review of the influenza A genome is in Appendix S1.

## Results

### Model Development and Description

I use a simplified model consisting of the major components of an immune response, which are epithelial cells, influenza A viruses, and an immune system, to describe the dynamics of an influenza A infection and the subsequent immune response. This model contains six state variables, which are the healthy cell concentration 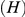, the infected cell concentration 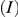, the viral load 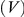, the concentrations of naïve and memory antibodies 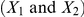, respectively, and the naïve antibody affinity 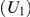. These state variables are in Table 1.

**Table 1:** Descriptions and units of the variables of the dynamical model.

Variable	Description	Unit
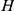	Healthy cell concentration	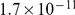 M
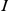	Infected cell concentration	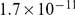 M
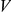	Viral load	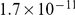 M
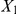	Concentration of naïve antibodies recognizing the virus	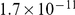 M
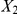	Concentration of memory antibodies from a previous infection or vaccination	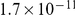 M
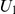	Affinity of naïve antibodies recognizing the virus	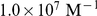
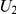	Affinity of memory antibodies from a previous infection or vaccination	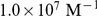
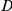	Dead cell concentration	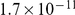 M

This model comes from the following information on influenza A infection. The concentration of epithelial cells on the upper respiratory tract is around a fixed homeostatic level 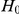, which is also the sum of the concentrations of healthy cells (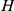), of infected cells (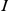), and of dead cells (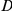) killed by the influenza A virus, respectively. Free influenza A virus particles (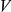) are released from infected cells and are eliminated by influenza A specific antibodies. I only consider virus clearance by antibodies, because a T cell can recognize influenza A strains with different antigenic characters [34]. This model has two types of antibodies: the naïve antibodies and the memory antibodies generated by the last influenza A infection or vaccination. The concentrations of the naïve and the memory antibodies are defined as 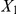 and 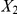, respectively. The immune system cleared the influenza A viruses bound by antibodies. With the definition of antibody affinity
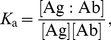
(1)the concentration of influenza A virus particles bound by antibodies 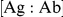 is proportional to the concentrations of the free influenza A virus particles 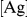 and of the influenza A specific antibodies 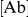. The naïve and memory antibodies have affinities 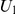 and 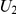, respectively, to the influenza A virus. The affinity of memory antibodies, 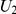, is a constant parameter of the model. The maximum affinity is defined as 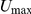. Here 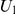 and 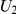 are quantified using the reduced unit 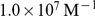, as described in the subsection Reduced Units and Parameter Estimation in the section Materials and Methods. Thus the affinities 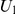 and 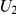 are defined as 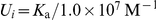 (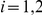). The maximum affinity is 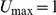 when 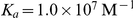.

From the above information, a minimal set of ODEs are built to model the influenza A specific immune response of co-existing naïve antibodies with a low initial affinity and memory antibodies with a higher and constant affinity. The state variables 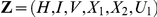 comprise the healthy cell concentration 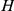, the infected cell concentration 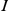, the viral load 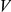, the naïve antibody concentration 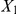, the memory antibody concentration 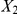, and the binding affinity of naïve antibodies 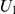.
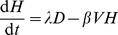
(2)

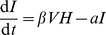
(3)

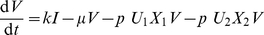
(4)

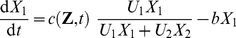
(5)

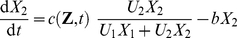
(6)

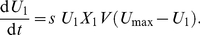
(7)The homeostasis of epithelial cells gives an additional algebraic equation for the dead cell concentration, 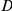,
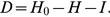
(8)


Equation 2 describes the dynamics of the concentration of healthy epithelial cells. The repair mechanism for epithelial tissue is activated only if any damage in epithelial cells is detected (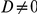), and new healthy cells are regenerated with the rate 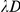 to repair the damaged tissue [25]. Alternative models for this repair mechanism include 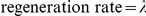 [28] or 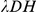 [29]. In the stochastic model in [32], the regeneration rate is 0 when 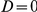 and has a mathematical expectation of 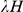 when 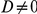. The average life span of human trachea cells is 47.5 days [35], while the time for epithelial cell regeneration is 0.3–1 day [25], [36], showing that the cell regeneration rate is significantly higher than the normal cell division rate. With this consideration in mind, I select the expression 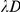 as the cell regeneration rate [25]. The infection rate 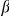 represents the speed in which influenza A virus converts healthy cells into infected cell. The protective effect of interferon is neglected in this simplified model.

Equation 3 characterizes the dynamics of the infected cell concentration. All the infected cells are converted from healthy cells. Infected cells are killed by the virus with the rate 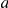.

Equation 4 depicts the generation and elimination of virus particles. Virus particles are released from infected cells with the rate 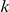. The half-life of free virus particles is 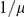. Viruses bound by antibodies are neutralized and cleared by the immune system. Thus the virus clearance rate is proportional to the concentration of viruses bound by antibodies 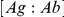. From equation 1, the virus clearance rate is proportional to the antibody affinity 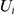 (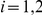), the antibody concentration 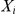 (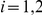), and the viral load 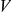, respectively.

Equations 5 and 6 show the secretion and decay of naïve antibodies and memory antibodies. Antigen presenting cells (APC) process the virus and present the antigen on their surface, activating naïve T cells. Some of these activated T cells proliferate and differentiate into Th2 helper T cells. Th2 cells and free virions activate B cells together [37]. The intensity of activation signal for B cells, 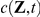, is a function of time depending on viruses, APCs, and naïve T cells. The intensity 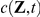 is a rectangular window function with the maximum value of 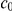, and is further described in the section Materials and Methods. Naïve B cells mature in germinal centers, undergoing proliferation and somatic hypermutation. B cells are selected by competing for antigen binding and activation signals from Th2 cells surrounding the germinal center. The morphology of germinal centers determines that the interface between the B cell region and the Th2 cell region is approximately constant, and so is the amount of antigens inside the germinal center. Therefore B cells inside the germinal center compete with each other for the activation signal. The ratio of the intensities of the activation signals for naïve and for memory B cells is 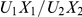. The decay rate of both naïve and memory antibodies is 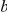. I use identical decay rate (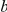) for naïve antibodies (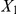) in equation 5 and memory antibodies (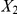) in equation 6 because the decay rate is independent of the type of antibodies [38].

Equation 7 indicates that the increase rate of the affinity is proportional to the concentration of the antigen-antibody complex. Because the B cells are selected by the affinity to the antigen in their maturation process, the increase of the naïve antibody affinity 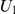 is driven by successful binding between the naïve antibody and the antigen. The logistic factor 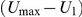 ensures that the probability for B cells to mutate to a state of higher affinity decreases as the maturation proceeds.

The dynamical model comprises equations 2–7. Equations 2, 3, 4, and 7 are adapted from previous models [24], [25], [29]. Note that equations 2, 3, 4, and 7 are not identical to their original form in literature. Most of these previous models were developed according to the processes of cell death and regeneration and virus entry and release. The parameters of these models come from experiment except for the parameter 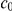, which describes the activation of B cells, and for the parameter 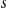, which describes the B cell maturation process. The parameter 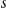 was fit to experimental data in a previous model [29]. As will be shown, I estimate the values of 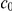 and 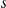 all over again and perform a sensitivity analysis to the parameters 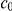 and 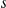.

The present six-ODE model is able to be mapped from a previous 10-ODE model developed by Hancioglu *et al.* [29]. Hancioglu *et al.*'s model contains 10 state variables, which are 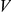 (virus), 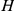 (healthy cells), 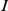 (infected cells), 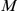 (antigen presenting cells), 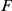 (interferons), 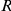 (virus resistant cells), 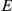 (cytotoxic T cells), 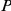 (plasma cells), 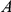 (antibodies), and 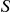 (antigenic distance between the antibody and the virus). Out of these 10 state variables, five variables 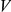, 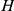, 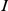, 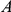, and 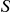 are also the state variables of my model, and the other five variables 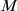, 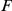, 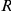, 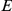, and 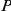 are incorporated into my model in a mean-field approach. In Hancioglu *et al.*'s model, state variables 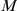 and 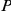 inhibit virus growth by increasing the concentration of antibody (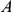), while state variables 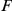, 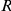, and 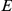 reduce the concentration of infected cells (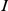) that produce virus. In my model, the intensity of activation signal for B cells is the term 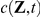 in equations 5 and 6. Compared to Hancioglu *et al.*'s 10-ODE model, my six-ODE model removes five state variables, while using two state variables 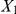 and 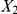 for the naïve and memory antibodies, respectively.

### Time Courses of Infection and Recovery

With all parameters defined and fixed in the section Materials and Methods, I use the stiff differential equation solver ode23s in MATLAB to numerically solve equations 2–7. The relative and absolute error tolerances of the solver are 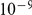 and 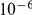, respectively. The first set of parameters listed in the left column of Table 2 are adopted. As described in the subsection Kinetics of Influenza A Virus Infection in the section Materials and Methods, at the moment of infection, all the epithelial cells are healthy cells, and the initial viral load is approximately 1% of the epithelial cell concentration. The concentration of naïve antibodies capable of recognizing the antigen is approximately 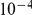 of the epithelial cell concentration, while the concentration of memory antibodies specific to the antigen is 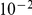 of the epithelial cell concentration. Initial affinity of naïve antibodies to the antigen is 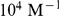, 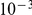 of the maximum affinity. Using the reduced units introduced in the section Materials and Methods, the initial values 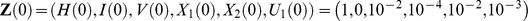. I run a simulation of 20 days. The solved trajectories of the state variables 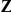 are compared to the kinetics of influenza A infection observed in reality to verify the model parameters.

**Table 2:** Parameters of the dynamical model.

	Physical meaning	Parameter	Parameter	Estimation	Unit
		set 1	set 2		
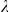	Regeneration rate of healthy epithelial cells	2			day 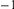
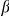	Infection rate	0.34	0.27		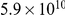 M 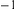 day 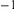
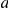	Death rate of infected epithelial cells	1.5	4.0		day 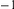
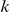	Rate of virus release from infected epithelial cells	510	480		day 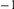
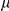	Nonspecific virus clearance rate	1.7	3.0		day 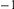
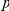	Rate of virus neutralization by antibodies	619.2			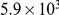 day 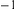
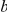	Decay rate of antibodies	0.043			day 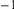
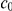	Production rate of antibodies			1.0	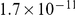 M day 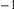
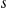	Maturation rate of B cells			100	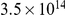 M 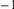 day 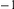

**Figure 1:**
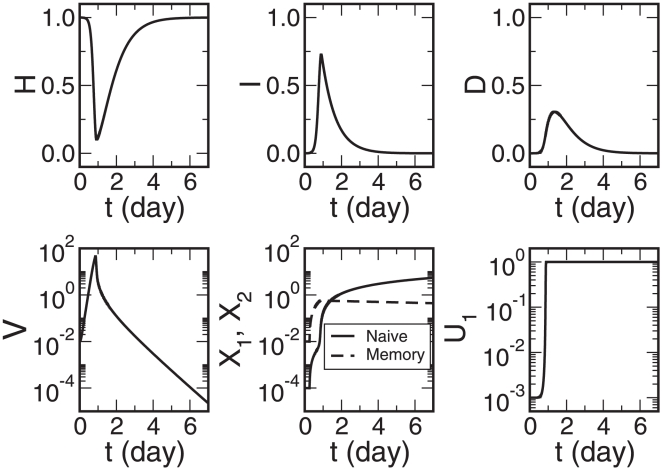
Time courses of the healthy cell concentration 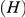, the infected cell concentration 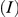, the dead cell concentration 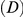, the viral load 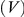, the concentrations of naïve and memory antibodies 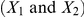, respectively, and the naïve antibody affinity 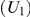. The memory antibody affinity is 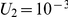. The initial conditions are 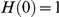, 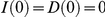, 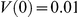, 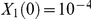, 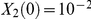, and 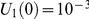.

**Figure 2:**
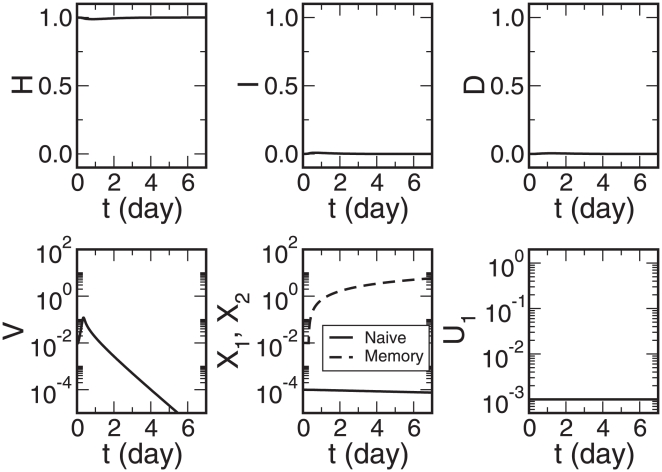
Time courses of the healthy cell concentration 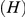, the infected cell concentration 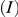, the dead cell concentration 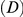, the viral load 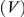, the concentrations of naïve and memory antibodies 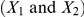, respectively, and the naïve antibody affinity 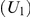. The memory antibody affinity is 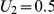. The initial conditions are the same as those in Figure 1.

I use two cases to illustrate the dynamics of all state variables. In the first case, the virus has substantially mutated from the previous strains, and the binding affinity of the memory antibodies to the virus is low. In the second case, there is no significant escape mutation of the virus, and the binding affinity of the memory antibodies to the virus is high. The affinity of memory antibodies is 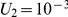 in the first case and is 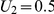 in the second case. The details of the model dynamics are shown in Figure 1 and 2.

Figure 1 describes the whole process of influenza A virus infection and clearance in humans without immune memory (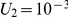). A symptom with approximately 30% of the epithelial cells killed is observed after the infection. The peak of the dead cell concentration 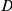 occurs on Day 1 to Day 2, agreeing with the experimental data [25]. On Day 5, the dead cell concentration 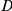 falls under 0.1. The viral load 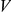 decreases to the initial level on Day 3 to Day 4. A 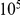-fold increase of the naïve antibody concentration occurs in the virus infection and clearance process. The naïve antibody affinity 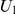 approaches to the maximum 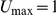. The memory antibody concentration 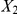 has an initial 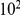-fold increase, and decreases approximately exponentially after Day 1 with the rate 0.0427, similar to the antibody decay rate 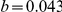. Thus few memory antibodies are produced after Day 1.

Figure 2 shows the dynamics of virus infection and clearance with immune memory (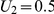). Few dead cells are accumulated and thus no symptoms are observable in the infected person. The viral load is remarkably suppressed compared to Figure 1. Figure 2 depicts the effect of a successful vaccination. Compared to Figure 1, the increase of the naïve antibodies concentration is absent, and the naïve antibody concentration, 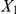, decreases approximately exponentially with the rate 0.0423, close to the antibody decay rate 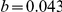, indicating that naïve antibodies are rarely produced during the whole process of virus infection and clearance. No significant somatic hypermutation is observed in those naïve B cells. The naïve antibody affinity 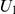 is almost constant, as shown by the plot of 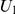 against 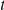. There is a notable increase in the memory antibody concentration 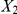: the value of 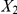 on Day 7 is approximately 10-fold higher than that in Figure 1.

The immune response is dominated by the naïve antibodies when the memory antibodies have low affinity and is dominated by the memory antibodies when they have high affinity. The transition between these two cases occurs when the value of the memory antibody affinity 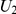 falls into a critical region. In the following subsection, 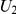 is set to a variety of values in the range 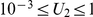. As the value of 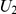 changes, the phenomenon of original antigenic sin can be observed in other characters of the dynamics, such as the maximum percentage of dead cells, the maximum viral load, the cumulative effects of naïve antibodies and of memory antibodies, and the average antibody affinity. This model is able to reproduce the phenomenon of original antigenic sin observed in the experimental data at intermediate memory antibody affinity 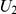.

### A General Picture of Original Antigenic Sin

**Figure 3:**
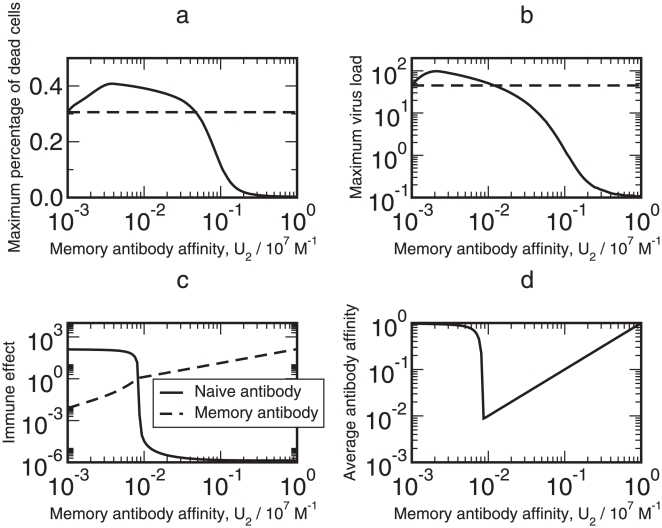
Trajectories of the maximum percentage of dead cells, the maximum viral load, the cumulative effects of naïve and memory antibodies defined by **equations 9** and **10**, respectively, and the final average antibody affinity defined by **equation 11** with different memory antibody affinities 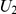. The dashed horizontal lines in (a) and (b) are the maximum percentage of dead cells and the maximum viral load, respectively, at the lowest memory antibody affinity 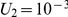.

To illustrate the phenomenon of original antigenic sin, I choose 100 values of 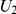 logarithmically spaced between 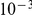 and 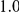. The minimum value 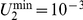 reflects the case that memory antibodies rarely recognize a new virus strain. The maximum value 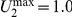 corresponds to the immune response with the highest memory antibody affinity. The intermediate values of 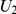 correspond to the case that memory antibodies have decreased capability to recognize a new virus strain due to the escape mutation of the strain or imperfect vaccination. One hundred independent simulations were run with these 100 values of 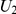, respectively. The maximum viral load and the maximum percentage of dead cells were recorded for each simulation. The cumulative effects of naïve antibodies and of memory antibodies are respectively calculated with
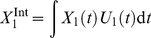
(9)

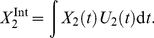
(10)Similarly, the average antibody affinity at the end of each simulation is
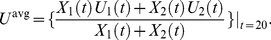
(11)Equation 11 calculates the average antibody affinity after the 20-day period of time during which the patient recovered from the infection. Note that indicated by equation 7, the naïve antibody affinity 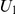 monotonically increases, while the memory antibody affinity 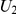 is constant. Figure 3 depicts the maximum percentage of dead cells, the maximum viral load, the cumulative effects of naïve and of memory antibodies, and the final average antibody affinity as the functions of the memory antibody affinity 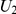.

Figure 3a presents a picture of original antigenic sin with the maximum percentage of dead cells. With 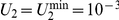, the maximum percentage of dead cells, 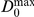, is 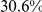. Original antigenic sin is observed in the interval 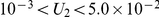. The peak in the figure, 40.8%, is reached when 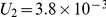 and is 33.3% higher than the maximum percentage 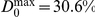 with 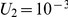. This 33.3% increase is significant for the percentage of dead epithelial cells. When 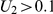, the maximum percentage of dead cells falls below 10%, indicating that no observable symptoms occur in the infected person. That is, when the antigenic distance from the previous infection or vaccination to the new virus strain is small, the immune system can clear the virus effectively.

Figure 3b shows the non-monotonicity of the maximum viral load 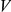 as a function of the memory antibody affinity 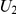 in the process of virus infection and clearance. The maximum viral load is 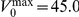 when 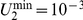. The maximum viral load in the region 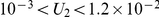 is higher than 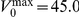, and so original antigenic sin occurs in this region. The maximum viral load in the interval 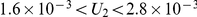 is at least twice as high as 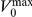. With 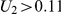, the maximum viral load is less than unity, agreeing with the fact that memory antibodies effectively recognize and eliminate virus strains which are antigenically similar to the immune memory [14].

Figure 3c describes the cumulative effects, 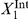 and 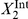, of naïve and memory antibodies in the process of virus infection and clearance, respectively. The cumulative effects 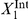 and 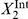 are calculated with equations 9 and 10, respectively. There is a threshold 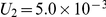 below which the cumulative effect of naïve antibodies 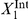 is in a plateau in which 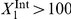. The variable 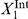 sharply decreases from 100 to 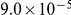 in a narrow region 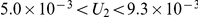. The variable 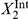 increases almost linearly with 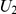 from 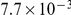 (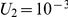) to 133.8 (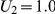). The variable 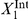 is larger than 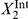 when 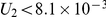 and is smaller otherwise. Because 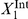 decreases quickly when 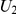 is near 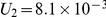, there is a large difference between 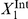 and 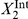 when 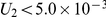 or 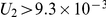. When 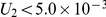, the naïve antibodies are the dominant type of antibodies which makes the major contribution to the clearance of influenza A virus. When 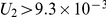, the memory antibodies are the dominant type.

Figure 3d plots the final average antibody affinity 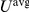 against the memory antibody affinity 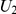. Equation 7 ensures the monotonic increase of 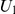, whereas the increase rate of 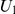 indirectly depends on 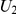. Similar to Figure 3c, a plateau with 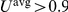 exists when 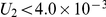, and 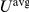 decreases substantially from 0.82 to 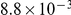 when 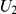 increases from 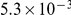 to 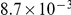. Note that in Figure 3c, this sudden decrease in the cumulative effect of naïve antibodies 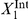 occurs in the same region of 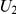. When 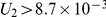, 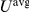 increases approximately linearly with 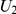, which is mainly due to the contribution of the memory antibodies.

### Mechanism of Original Antigenic Sin

**Figure 4:**
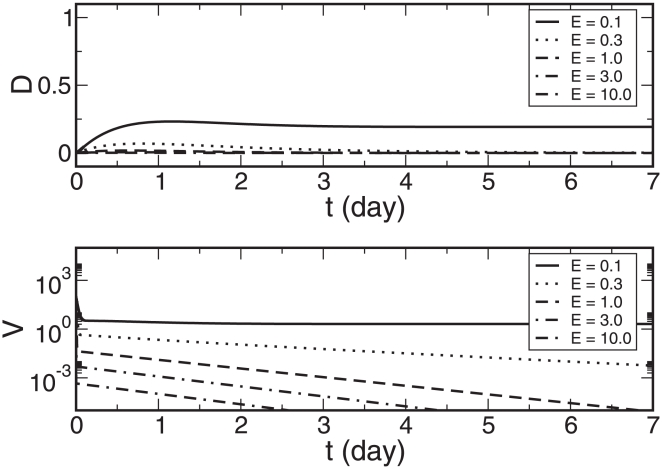
Trajectories of the concentration of dead cells 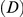 and the viral load 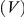 with different effects of immune response 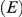. In each trajectory, 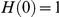, 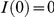, 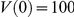, and 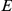 is constant. When 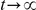, viruses cannot be cleared at small values of 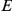, such as 0.1. The decay rates of both 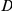 and 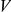 increase with 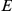.

The dynamical system defined by equations 2–7 can be split into two subsystems, i.e. an actuator and a controller, with weak coupling between them. Equations 2–4 constitute the actuator with 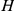, 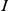, and 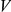 as the state variables. Equations 5–7 constitute the controller with 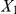, 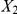, and 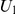 as the state variables. The actuator is controlled by the variable 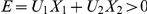. Therefore equation 4 is equivalent to
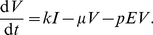
(12)The actuator consisting of equations 2, 3, and 12 has two steady states:
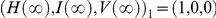


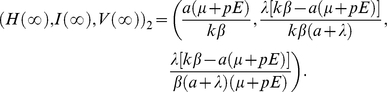
By calculating the eigenvalues of the Jacobian of the actuator, I find that the first steady state is stable for any 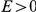, and the second one is stable only with 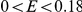. When the immune response is strong, 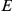 is large and the only steady state, 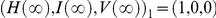, indicates a complete recovery from the infection. In the process of virus infection and clearance, a large value of 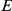 ameliorates the infection of healthy cells by suppressing virus proliferation. The numerical simulation of the actuator illustrates the dependence on 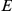 of the dynamics of influenza A infection. Figure 4 displays the effect of 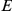: all the viruses are cleared from the patient when 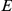 is larger than 0.18, and the decay rates of the dead cell concentration 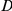 and of the viral load 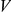 increase with 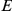. Therefore, 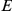 is the only factor controlling the values of 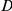 and 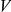.

**Figure 5:**
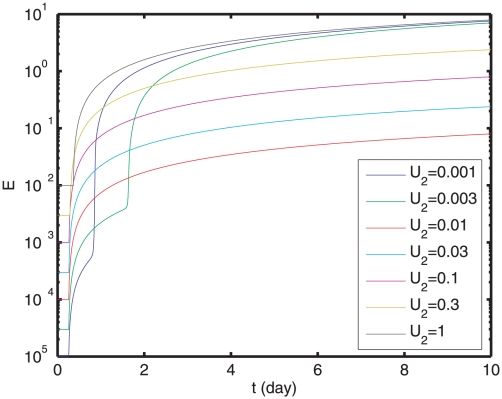
Trajectories of the effect of immune response, 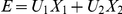. In each trajectory, 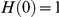, 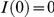, 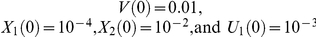. Each trajectory corresponds to one value of 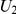. When 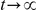, viruses cannot be cleared at small values of 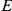, such as 0.1.

The other subsystem is the controller comprising the state variables 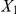, 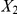, and 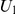. The controller observes the state of the actuator as the factor 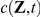, which jumps from 0 to 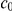 when the viral load 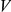 reaches 0.1 and remains 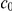 for 14 days. Due to the quick virus proliferation at the beginning of the infection, I let 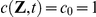 as an approximation. The dynamics of the expression 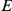 are described by the following equation:
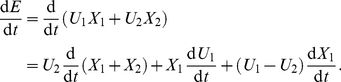
(13)The first term on the right hand side of equation 13, 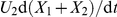, is the product of 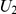 and the derivative of a first order process 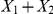. The derivative of 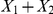 is obtained by adding equation 5 to equation 6 with the approximation 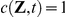 and is independent of 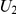. The form of equations 5 and 7 shows that 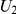 suppresses the variables 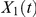 and 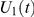, and so the term 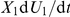 monotonically decreases with 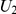. In the case of small 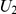, the factor 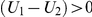 in the process of virus infection and clearance (see Figure 1), thus the third term 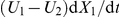 decreases with 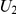. In the case of large 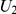, 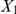 is approximately constant, and the third term is negligible. Consequently, the first term in equation 13 increases with 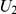 and the other terms decrease with 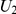. Figure 5 shows 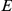 as a functional of 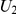: when 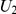 increases from 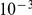 to 1, the increases in the second and third terms of 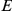 do not compensate the decrease in the first term, yielding a suppression of 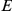 at intermediate 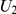.

The source of original antigenic sin is the interaction of the state variables of the controller, or the immune system, modeled by equations 5–7. When the memory antibody affinity 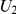 is either low or high, naïve or memory antibodies are dominant, respectively. If naïve antibodies are dominant, their final affinity is high. At intermediate 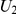, the interaction and competition between naïve and memory antibodies lead to a decreased immune effect 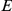, which clears the viruses less effectively. This is original antigenic sin. When original antigenic sin occurs, the influenza illness rate increases [14] due to the increases of 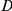 and 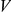 during the process, and the average antibody affinity decreases [17] due to the decrease of 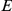. Note that the average antibody affinity is defined as 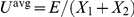, where 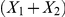 are approximately independent of 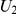, as discussed above.

### Sensitivity Analysis

The parameters other than 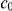 and 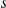 come from literature. See Table 2. As mentioned in the subsection Model Development and Description, my model described by equations 2–7 can be mapped from a previous dynamical model of influenza infection and immune response [29], in which a comprehensive sensitivity analysis for parameters is available. The sensitivity analysis for most of the parameters in this model has been performed in [29]. So I first focus on two remaining parameters, 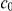 and 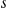. The parameter 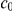 characterizes the stimulation of the immune system when the viral load increases beyond a threshold. Because this paper aims to give a simplified model comprising the most important factors of both epithelial cells and an immune system, the effects of APC and Th2 cells are combined into the parameter 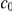. The parameter 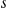 reflects the process of B cell somatic hypermutation which produces antibodies with high affinity to the antigens. Here I present a sensitivity analysis for the parameters.

**Figure 6:**
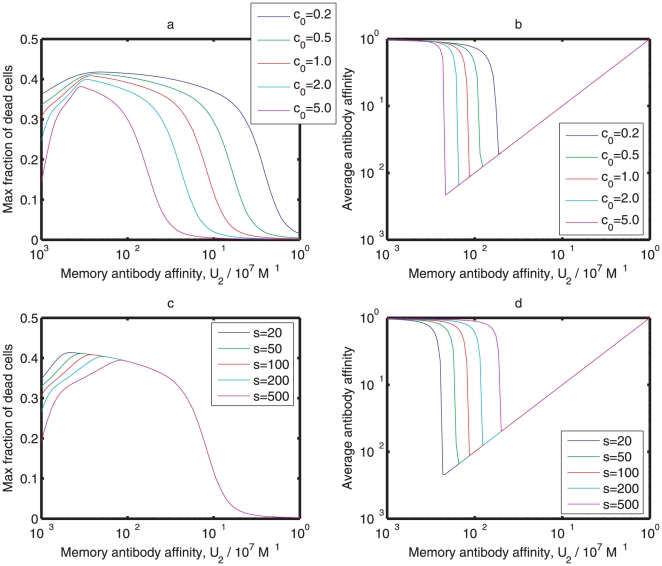
Sensitivity analysis of parameters 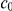 and 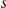. (a) and (b) The maximum percentages of dead cells and the average antibody affinities at different values of 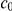. (c) and (d) The maximum percentages of dead cells and the average antibody affinities at different values of 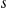. Initial conditions and parameters other than 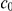 and 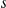 are the same as those in Figure 3.

**Figure 7:**
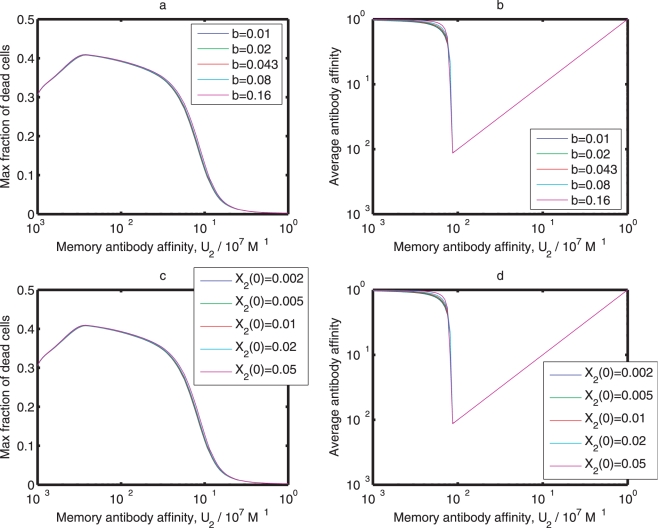
Sensitivity analysis of parameters 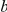 and 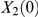. (a) and (b) The maximum percentages of dead cells and the average antibody affinities at different values of 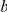. (c) and (d) The maximum percentages of dead cells and the average antibody affinities at different values of 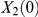. Initial conditions and parameters other than 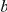 and 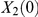 are the same as those in Figure 3.

Figure 6 describes the behavior of the dynamical system with different values of parameters 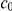 and 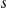. The maximum percentage of dead cells with large 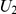 is insensitive to 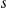. The average antibody affinity with small and large 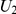 is also insensitive to both 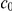 and 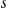, but is sensitive to intermediate 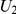. If 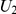 is higher than a threshold, the memory antibodies play the major role in the immune response; otherwise the naïve antibodies mainly conduct the immune response. As shown in Figure 6b and 6d, this threshold of 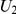 decreases with 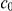 and increases with 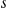. However, the dynamics with different values of 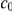 and 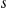 in Figure 6 resemble those in Figure 3a and 3d. The existence of original antigenic sin is insensitive to the parameters 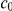 and 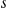. In addition, Figure 7 shows that both the maximum percentage of dead cells and the average antibody affinity 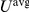 are insensitive to the decay rate of antibodies (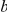) and the initial concentration of memory antibodies (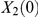). This sensitivity analysis shows that the severity of an influenza A infection decreases with 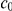, and the effect of original antigenic sin increases with 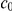 and decreases with 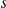.

## Discussion

The model defined by equations 2–7 is a significant simplification to the previous models describing the kinetics of influenza A infection [25], [29]. This model introduces a second type of antibodies, the memory antibodies, to simulate the competition and cooperation between naïve and memory antibodies. The contributions of Th1 cells, cytotoxic T lymphocytes (CTLs), interferons, and epithelial cells protected by interferons are incorporated into the parameters of the model. The contributions of APCs and Th2 cells, which together activate B cells, are captured by the factor 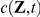 in a mean-field approach, rather than being explicitly modeled using ODEs. The presenting model has two limitations. First, at the same time of the increase in the dead cell concentration 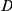, the viral load 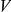 increases by 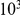
–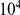 fold and reaches the maximum on Day 1, which is different from the experimental results of 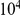
–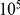 fold increase at the maximum on Day 2. Second, this model does not contain a term modeling the loss of antibodies due to virus binding in equations 5 and 6. These limitations are due to simplification of the model. However, these limitations do not seriously affect the emergence of original antigenic sin in the model at intermediate memory antibody affinity 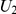, the major topic of this study.

The concentration of CTLs increases by 100 times in the first seven days after infection [25] to eliminate the infected cells. The cellular immune system usually has strong cross immunity for antigenically different influenza A virus strains [25], while the humoral immune system cannot effectively recognize a new influenza A virus strain with a large antigenic distance from the previous strains seen by the host immune system [14]. Thus the effects of CTLs against different influenza A virus strains are more homogeneous than those of antibodies. That is, CTLs induced by previous virus strains can effectively suppress a new virus strains despite the escape mutation of the virus [25]. By contrast, the antibody affinity decreases with the antigenic distance between the previous virus strains and the new strain. Thus the contribution of CTLs is more constant compared to that of antibody and can be modeled by constant parameters to describe original antigenic sin.

For the same reason, the protection for healthy cells by the interferon secreted by infected epithelial cells is not modeled as an independent equation either. Additionally, the interferon and the cells protected by the interferon are not the key factors of the dynamics of virus infection and clearance: an absence of interferons does not affect the final elimination of all viruses and dead cells [29]. APCs and Th2 cells have little interaction with the elements in the model other than the antibodies. Hence a simple function 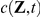 is introduced to model the activation of B cells.

The present model contains two parts. Equations 2–4 constitute a general model for an infection in tissue caused by a cytopathic virus. Equations 5–7 define a model for the immune system which recognizes and clears the virus. This model can be extended to include the dynamics of CTLs and interferons in the immune system. The model can also be extended to simultaneously consider multiple types of cytopathic viruses by using equations 2–4 with different parameter sets 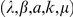 to model each type of virus. The cytopathic viruses fall into two categories: those causing acute diseases and those causing chronic diseases. Viruses in the first category, such as influenza A virus, are cleared in a short period of time, thus relatively few escape mutations occur. The immune system therefore directs itself towards a fixed target. Equations 5–7 modeling the immune system do not require any modification to take into account the escape mutation of the virus. On the other hand, viruses in the second category, including HIV, persist for years in the host and keep mutating away from the immune system. A new set of ODEs are needed to model this case. First, additional terms are required for equation 7 to describe the decrease of the memory antibody affinity due to the escape mutation of the virus. Second, if the immune system is also infected by the virus, equations 5 and 6 should also contain terms to model this infection. A similar model of the immune response against HIV and the competition between antibodies has been developed [39].

The currently available mathematical models of original antigenic sin falls into two categories: stochastic models represented by the GNK model [17], and deterministic models as the present one. Now I compare the mathematical form of the GNK model [17] with that of my deterministic model. Both models consider the contributions of naïve and memory B cells. Both models explicitly simulate the competition between different types of B cells. In the deterministic model, the maturation of naïve B cells follows a logistic process. In the GNK model, the B cells have random walks on a rugged and random landscape [17], [19] where the density of neighboring states with higher affinities decreases with the affinity in the current state. The deterministic model has two variables, 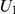 and 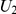, for the naïve and memory antibody affinities, respectively. The GNK model, however, stores the amino acid sequences of 1000 naïve antibodies and other 1000 memory antibodies [17]. After a simulation of 30 generations, the number of different amino acid sequences generally converges to less than five, similar to the deterministic model in which both naïve and memory antibodies are considered as monoclonal.

There are parallels and differences between my deterministic model and the GNK model [17], although these two models have different mathematical forms as shown above. First, the deterministic model uses the factors 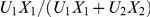 and 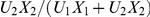 to model the selection of naïve and memory B cells, respectively. In an immune response, B cells producing antibodies with high binding affinity 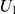 or 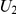 are selected. As a comparison, the stochastic model stores the amino acid sequence of each B cell, selects those B cells with high affinity to the antigen, and replicates the selected B cells to the next generation. Second, the deterministic model simulates the B cell maturation process with the factor 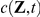 describing the activation of B cells. In the simulation, 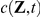 is greater than zero for 14 days, in which the B cells compete for the antigen and divide. The stochastic model repeats the process of B cell hypermutation and selection for 30 generations of B cells, which correspond to the primary or secondary immune response. Third, the naïve antibody affinity in the deterministic model, 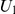, is modeled by equation 7, a logistic equation. The increase rate of 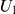 decreases as 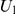 approaches to the maximum antibody affinity, 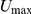. The stochastic model builds a rugged antibody affinity landscape, in which the locations with high affinities have low density. Consequently, the deterministic model is a mean-field approximation of the B cell maturation process. The deterministic model is able to simulate original antigenic sin with reduced memory and CPU requirement, while ignoring the amino acid sequence of each B cell.

The dynamical model introduced in this paper is deterministic, while the process of influenza A infection and clearance is stochastic in nature. However, the deterministic model gives similar results as the stochastic models [16], [17]. The deterministic model assumes both naïve and memory antibodies to be monoclonal. If either naïve or memory antibodies have multiple amino acid sequences, the ODEs could be modified by introducing more types of antibodies. Hence the competition factors 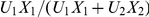 and 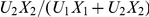 could be replaced by a tournament-like algorithm involving all the antibodies recognizing the antigen. By introducing the method of splitting the dynamical system into an actuator and a controller, the present model provides a starting point for the application of nonlinear control theory to explain original antigenic sin. The dynamical model can also be helpful to rational vaccine design.

## Materials and Methods

### Kinetics of Influenza A Virus Infection

Influenza A virus infection can be described by a dynamical process. The infection occurs in the epithelial cells on the surface of upper respiratory tract in the bronchi with diameter larger than 3.3 mm [25]. The incubation period between the infection and the emergence of symptoms ranges from one day to five days and is typically two days. The host starts to shed infectious virus particle approximately 24 hours prior to the emergence of symptoms. The typical initial concentration of influenza A virus particles is 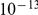 M. The viral load usually reaches a maximum 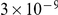 M two days after infection and falls back to the initial level six days after infection [25]. Influenza A virus is cytopathic and kills the infected cells, causing the dead cells to accumulate *in situ*. The percentage of dead epithelial cells reaches the maximum of 30–50% on Day 2 and decreases to 10% on Day 5. If the maximum percentage is lower than 10% in the process of virus infection and clearance, no symptoms are observable [25]. The immune system is activated by the detection of virus particles. The most important suppressor of influenza A virus are antibodies IgG and IgA, followed by the CD8 CTLs [37]. The concentrations of B cells and of plasma cells increase by 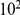 times and 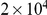 times, respectively, within seven days [25]. The immune response to a primary infection generates memory antibodies with binding affinity 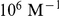 and concentration 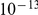 M, constituting 0.1%–1% of the total antibodies [29], [37]. The naïve antibodies capable of recognizing the antigen have the affinity 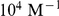 and concentration 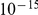 M [29], [37], constituting 0.001%–0.01% of the total antibodies.

### Reduced Units and Parameter Estimation

I use reduced units for all the variables and parameters to make their values close to unity and to facilitate the numerical calculation. For the state variables 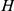, 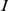, 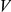, 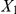, and 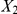, the unit is defined as the homeostatic concentration of epithelial cells in the upper respiratory tract, which is 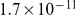 M [25], [29]. The unit of 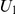 and 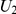 is defined as the maximum affinity between memory antibodies and influenza A viruses, which is 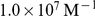 [37]. The reduced units for all the variables in equations 2–7 are listed in Table 1.

The majority of the parameters in this dynamical model are obtained from previous experiments. These parameters fall into two sets that are compatible with each other. The first set of parameters were given by the publications [24], [25], [29], [36]. These publications depicted the process of influenza A virus infection and clearance in the cellular level, taking into account the concentrations of epithelial cells, viruses, APCs, interferons, Th1 and Th2 helper cells, CTLs, B cells, plasma cells, and antibodies. The second set of parameters were extracted from an influenza A virus infection experiment with six volunteers [27]. A simpler ODE model with a fixed parametric form was built to fit the daily viral loads data measured from nasal wash [27]. These two sets of parameters in the reduced units are listed in Table 2. Despite the different approaches to obtain the parameters, the parameters 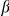, 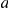, 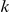, and 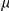 from [24], [25], [29], [36] and [27] are similar.

Compared to some previous models [25], [29], a major simplification in this study is neglecting the propagation of the activation signal for the immune system, originated by the detection of the virus and through APCs, Th2 cells, and B cells. Instead, I introduce a time-dependent factor 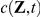 to model the activation signal for the immune system in a mean-field approach. In a typical process of influenza A virus infection, the viral load and the concentration of APCs reach the maximum simultaneously on Day 2 [25]. The viral load decreases to the initial level on Day 6 [25]. As listed in Table 3, the half-lives of APCs, helper T cells, B cells, and plasma cells are similar to the duration of the infection. Thus the duration of B cell maturation process is estimated to be 14 days, longer than the duration of viral clearance. Accordingly, the factor 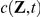 has the initial value of zero, is assigned the value 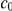 when 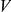 reaches 0.1, and equals to 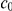 for 14 days before decreasing to zero again. Using the output of the previous models [25], [29], I estimate the parameter 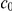 to be 1.0, and the parameter 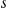 to be 100.

**Table 3:** Decay rates of different immune cells.

Immune cell	Decay rate/ 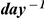	Reference
APC (in the stimulated state)	1	[29]
Macrophage	1	[25]
Th1 helper cell	1	[25]
Th2 helper cell	1	[25]
B cell	0.1	[25]
Plasma cell	0.4	[25]

## Supporting Information

Appendix S1 **A review of the Influenza A genome.**
(PDF)
