# Supplementary material for: Understanding Original Antigenic Sin in Influenza with a Dynamical System
Source: PLoS One. 2011 Aug 29;6(8):e23910. doi: 10.1371/journal.pone.0023910 (PMC3163660; doi:10.1371/journal.pone.0023910)
Supplement: Appendix S1 — A review of the Influenza A genome. (PDF) [file pone.0023910.s001.pdf]

Understanding Original Antigenic Sin in Influenza with a Dynamical System

Keyao Pan[1,*]
1 Department of Bioengineering, Rice University, Houston, Texas, USA
∗ **E-mail: keyaop@gmail.com**

Appendix

A Review of the Influenza A Genome

In this paper, I model the influenza A virus infection to study the mechanism of original antigenic sin. Influenza A virus is an RNA virus in the family Orthomyxoviridae. A functional influenza A virus particle consists of a spherical lipid bilayer shell and eight distinct RNA molecules which encode 11 proteins. Two glycoproteins, hemagglutinin (HA) and neuraminidase (NA), are on the surface of the virus particle. HA contributes to the binding of the virus to the sialic acid on the surface of the target upper respiratory tract epithelial cells and facilitates subsequent virus fusion and entry into the cells [1–3]. NA is the key component that facilitates virus release from the surface membrane of infected cells [4]. Nucleocapsid protein (NP), a nucleoprotein, encapsulates and transports viral RNA in the host cells [5]. The matrix protein, M1, binds to the inner side of the viral lipid bilayer membrane, helps assemble virus particles in the host cells, and is the central component in virus budding [4]. M2, a glycoprotein inside the lipid bilayer, serves as the ion channel adjusting the pH value in the virus particle, uncoats the virus particle, and contributes to efficient virus replication [6]. Additionally, there are two non-structural proteins, namely NS1 and nuclear export protein (NEP, formerly named NS2), as well as four RNA polymerases, namely PA, PB1, recently discovered PB1-F2 [7], and PB2. NS1 interferes with the cellular antivirus defense system [8]. NEP exports newly synthesized viral ribonucleoprotein (RNP) complexes comprising viral RNA, NP, and PB1 from the nucleus [9]. PA, PB1, PB1-F2, and PB2 replicate the viral RNAs in the nucleus of the infected cell.

References

1. Wilson IA, Cox NJ (1990) Structural basis of immune recognition of influenza virus hemagglutinin. Annu Rev Immunol 8: 737–771.

2. Skehel JJ, Wiley DC (2000) Receptor binding and membrane fusion in virus entry: the influenza hemagglutinin. Annu Rev Biochem 69: 531–569.

3. Frank SA (2002) Immunology and evolution of infectious disease. Princeton, N.J.: Princeton University Press.

4. Nayak DP, Hui EKW, Barman S (2004) Assembly and budding of influenza virus. Virus Res 106: 147–165.

5. Portela A, Digard P (2002) The influenza virus nucleoprotein: a multifunctional RNA-binding protein pivotal to virus replication. J Gen Virol 83: 723–734.

6. Takeda M, Pekosz A, Shuck K, Pinto LH, Lamb RA (2002) Influenza A virus M_2 ion channel activity is essential for efficient replication in tissue culture. J Virol 76: 1391–1399.

7. Chen WS, Calvo PA, Malide D, Gibbs J, Schubert U, et al. (2001) A novel influenza A virus mitochondrial protein that induces cell death. Nat Med 7: 1306–1312.

8. Li SD, Min JY, Krug RM, Sen GC (2006) Binding of the influenza A virus NS1 protein to PKR mediates the inhibition of its activation by either PACT or double-stranded RNA. Virology 349: 13–21.

9. O'Neill RE, Talon J, Palese P (1998) The influenza virus NEP (NS2 protein) mediates the nuclear export of viral ribonucleoproteins. EMBO J 17: 288–296.
